# Dysregulated Mitochondrial Dynamics and Metabolism in Obesity, Diabetes, and Cancer

**DOI:** 10.3389/fendo.2019.00570

**Published:** 2019-09-03

**Authors:** Wenting Dai, Lei Jiang

**Affiliations:** ^1^Department of Molecular and Cellular Endocrinology, Diabetes and Metabolism Research Institute, Duarte, CA, United States; ^2^Comprehensive Cancer Center, City of Hope Medical Center, Duarte, CA, United States

**Keywords:** energy metabolism, TCA cycle, mitochondrial dynamics, GTPase, mdivi-1

## Abstract

Metabolism describes the life-sustaining chemical reactions in organisms that provide both energy and building blocks for cellular survival and proliferation. Dysregulated metabolism leads to many life-threatening diseases including obesity, diabetes, and cancer. Mitochondria, subcellular organelles, contain the central energy-producing metabolic pathway, the tricarboxylic acid (TCA) cycle. Also, mitochondria exist in a dynamic network orchestrated by extracellular nutrient levels and intracellular energy needs. Upon stimulation, mitochondria undergo consistent interchange through fusion (small to big) and fission (big to small) processes. Mitochondrial fusion is primarily controlled by three GTPases, mitofusin 1 (Mfn1), Mfn2, and optic atrophy 1 (Opa1), while mitochondrial fission is primarily regulated by GTPase dynamin-related protein 1 (Drp1). Dysregulated activity of these GTPases results in disrupted mitochondrial dynamics and cellular metabolism. This review will update the metabolic roles of these GTPases in obesity, diabetes, and cancer.

## Introduction

The word *mitochondrion* comes from the Greek words *mitos* (*thread* in English) and *chondrion* (*granule* in English) (1). The field of mitochondrial dynamics has a history of over 100 years (1–3). In 1914, Mrs. and Mr. Lewis published “Mitochondria in Tissue Culture” as a special article in *Science* (2) and stated, “The mitochondria are almost never at rest, but are continually changing their position and also their shape.” The full picture of mitochondrial dynamics includes fusion, fission, transportation, and degradation, which have been covered in other reviews (1, 4–6). Mitochondria exist widely in most eukaryotic organisms and have a double membrane structure. The outer mitochondrial membrane contains large quantities of integral membrane proteins called porins, which form channels allowing for small molecules to freely diffuse from one side of the membrane to the other. In contrast, the inner mitochondrial membrane is highly impermeable, even to small metabolites. Three mitochondrial membrane–located GTPases regulate the mitochondrial fusion process, among which Mfn1 and Mfn2 control outer mitochondrial membrane fusion (7), while Opa1 regulates inner mitochondrial membrane fusion (8–10). In contrast, the cytosol-located GTPase Drp1 mediates the mitochondrial fission process (11). Mutations of these GTPases are closely related to human disease, such as Charcot–Marie–Tooth disease type 2A (12) and optic atrophy (13, 14). In this review, we primarily focus on these small GTPases and their influences on metabolic alterations in obesity, diabetes, and cancer.

## SMALL GTPases Control Mitochondrial Fusion and Fission

Mfn1 and Mfn2, homologs of the drosophila protein fuzzy onion (Fzo) (15), are the first reported mammalian GTPases to mediate mitochondrial fusion (16, 17). Mice lacking Mfn1 or Mfn2 are embryonically lethal, which suggests their essential role in embryonic development (7, 18). In mouse embryonic fibroblasts (MEFs), Mfn1 or Mfn2 single-knockout results in fragmented mitochondria (7), while Mfn1 and Mfn2 double-knockout completely blocks mitochondrial fusion (18, 19). Conversely, overexpression of either Mfn1 or Mfn2 can rescue the mitochondrial fusion (7). These genetic studies show that Mfn1 and Mfn2 have redundant and collaborative roles in mitochondrial fusion.

Opa1 is named after a neurogenic disease optic atrophy. Patients with mutant Opa1 have degenerated retinal ganglion cells and atrophy of the optic nerve (13, 14). Opa1 is the mammalian ortholog of Mgm1, which is essential for mitochondrial fusion in yeast (20, 21). Opa1 is encoded by a complicated set of at least eight mRNA splice forms, and each long Opa1 forms (L-Opa1) peptides that can be further cleaved into short forms (S-Opa1) by proteases Yeast Mitochondrial Escape 1 Like 1 ATPase (YME1L1) and metalloendopeptidase Oma1 (22, 23). YME1L1 and Oma1 double-knockout MEFs only contain L-Opa1 (24). Importantly, YME1L1 and Oma1 establish a near-balance of L-Opa1 and S-Opa1 under basal conditions, and they regulate this balance in response to metabolic changes or mitochondrial dysfunction (24–26). Moreover, the distinction between L-Opa1 and S-Opa1 lies in their different locations within mitochondria, namely, L-Opa1 in the inner mitochondrial membrane and S-Opa1 within the mitochondrial matrix. Consistent with their respective locations, L-Opa1 promotes mitochondrial inner membrane fusion, while S-Opa1 enhances mitochondrial fission (27).

Drp1, a cytosolic protein, plays a crucial role in regulating mitochondrial fission (4). A newborn girl with a dominant-negative mutation in Drp1 had abnormal brain development and several other diseases, which were associated with a severe defect in the fission of both mitochondria and peroxisomes (28). Drp1 knockout mice also exhibit abnormal brain development and die around day 12 of embryonic development (29, 30). Upon activation, Drp1 relocates to the outer mitochondrial membrane and forms a ring structure to constrict mitochondrion. This translocation of Drp1 is regulated by several outer mitochondrial membrane proteins, including fission protein 1 (Fis1), mitochondrial fission factor (Mff), and mitochondrial dynamics proteins of 49 and 51 kDa (MiD49 and MiD51) (31, 32). In mammals, overexpression of Fis1 in mammalian cells promotes mitochondrial fission, while inhibition of Fis1 results in elongation (fusion) (33–35). Knockdown of Mff results in mitochondrial elongation in mammalian cells (35) and reduces the amount of Drp1 recruited to mitochondria (36). In 2011, Palmer et al. reported that a double knockdown of MiD49 and MiD51 leads to mitochondrial elongation and reduces recruitment of Drp1 to mitochondria (37). Subsequently, MiD49 and MiD51 proteins were found to promote mitochondrial fission independent of both Fis1 and Mff (31, 38). Furthermore, the activity of Drp1 could be controlled by its phosphorylation. The cyclin-dependent Drp1 phosphorylation at serine 585 (S585) is essential to promote mitochondrial fission in mitotic cells (39). The cyclic adenosine monophosphate (cAMP)-dependent protein kinase (PKA)–dependent Drp1 phosphorylation at S656 inhibits mitochondrial fission, and the calcineurin-dependent dephosphorylation of Drp1–S656 promotes mitochondrial fragmentation (40). Furthermore, mitogen-activated protein kinase 1 (MAPK1, also known as Erk2)–dependent Drp1 phosphorylation at S616 activates Drp1 and promotes mitochondrial fission (41, 42). In HeLa cells, Drp1 was found to be phosphorylated by protein kinase A (PKA) at S637, and this phosphorylation attenuated the Drp1 GTPase activity (43). Besides, calcineurin-dependent dephosphorylation of Drp1 at the S637 site drives its translocation to mitochondria and promotes mitochondrial fission (44). Genetical and chemical inhibition of Drp1 both result in elongated mitochondria (45).

## Imbalanced Mitochondrial Metabolism Results in Obesity and Diabetes

Given the essential role of mitochondria in energy metabolism, mitochondrial dysfunction acts as a key regulator in the pathophysiology of obesity and diabetes. Mutations in some mitochondrial genes have been demonstrated to be the main causes of these metabolic diseases, such as Charcot–Marie–Tooth disease (Mfn2 mutation) and dominant optic atrophy (Opa1 mutation) (4). Interestingly, imbalanced mitochondrial metabolism induced by mitochondrial proteins (especially mitofusins, Opa1, and Drp1) in various tissues has been implicated in the pathology of metabolic diseases (Table 1).

**Table 1:** The role of GTPases in obesity and diabetes.

**GTPase**	**Alterations in expression**	**Tissue or cell**	**Mitochondrial metabolism**	**References**
Mfn2	Reduced expression in obese subjects and T2D patients	Skeletal muscle cells	Reduce OXPHOS	(46, 47)
	Mfn2 knockout rat	Skeletal muscle cells	Reduce OXPHOS	(48)
	Mfn2 overexpression rat	Skeletal muscle cells	Enhance OXPHOS	(48)
	Mfn2 knockout mice	POMC neurons	Reduce OXPHOS	(49)
	Mfn2 knockout mice	Liver	Reduce OXPHOS and respiratory rate	(50)
	Mfn2 knockout mice	Brown adipose tissue	Reduce OXPHOS but enhance fatty acid oxidation in female mice	(51, 52)
Mfn1	Reduced expression in diabetic patients	Heart and myocardial	Reduce OXPHOS in glucose utilization but increase fatty acid utilization	(53)
	Mfn1 knockout mice	Liver	Enhance lipid oxidation but not glucose oxidation	(54)
	Reduced expression in male mice	Skeletal muscle cells	Reduce OXPHOS	(55)
	Mfn1 knockout mice	Brown adipose tissue	No effect on energy expenditure	(51)
	Mfn1 knockout mice	POMC neurons	Reduce OCR and energy expenditure; defective insulin secretion and abnormal glucose homeostasis	(56)
Opa1	Opa1 knockdown cell	Adipose tissue and 3T3-L1 adipocytes	Reduce lipolysis and glycerol release by regulating phosphorylation of perilipin 1	(57)
	Opa1 knockdown mice	Skeletal muscle cells	Reduce OXPHOS and destabilize the respiratory chain supercomplexes	(58)
	Opa1 knockdown mice	Skeletal muscle cells	Reduce OXPHOS	(59)
	Opa1 knockout mice	Skeletal muscle cells	Impair mitochondrial respiratory capacity and disrupt lipid metabolism	(60)
	Higher expression in *ad libitum* feeding dog	Adipose tissue	Enhance OXPHOS	(61)
	Opa1 knockout mice	Pancreatic β cells	Reduce OXPHOS, glucose-stimulated ATP production, and insulin secretion	(62)
	Opa1 knockdown cell	Cardiomyocyte	Reduce OXPHOS and intracellular ATP levels	(63)
Drp1	Overexpression of Drp1	Skeletal muscle cells	No deficiency in mitochondria bioenergetics	(64)
	Deletion of Drp1	Skeletal muscle cells	Enhance oxidative metabolism and uncoupling of OXPHOS	(65)
	Decrease in Drp1 S616 phosphorylation in obese subjects after exercise or in mice	Skeletal muscle cells	Reduce ATP production but increase fat oxidation and insulin sensitivity	(66)
	Drp1 knockdown genetically or pharmacological inactivation by mdivi-1	Human and mouse pancreatic β cells	Not directly impair glucose-stimulated OXPHOS, but instead limit mitochondrial ATP synthesis by compromising substrate delivery upstream of mitochondria	(67)
	Drp1 pharmacological inactivation by mdivi-1	Diabetes-susceptible cybrid cells	Enhance OXPHOS and insulin-mediated glucose uptake	(68)
		3T3-L1 adipocytes	Not affect ROS production, lipid accumulation, or lipid metabolism	(68)
	Drp1 knockout mice	Pancreatic β cells	Normal OCR, but significantly reduce second-phase insulin secretion and glucose-stimulated amplification of insulin secretion	(69)
	Drp1 knockout mice	Skeletal muscle cells	Enhance insulin signaling and systemic insulin sensitivity	(65, 66, 70)
		Skeletal muscle cells	Reduce activities of complexes I and III	(71)
		Liver	Enhance energy expenditure	(72)
		POMC neurons, dorsal vagal complex	Increase ROS content, leptin sensitivity, and glucose responsiveness	(73, 74)
	Overexpression of Drp1	Diabetes-susceptible cybrid cells	Increase mitochondrial ROS; reduce IRS1–AKT pathway and GLUT translocation	(75)

*OXPHOS, oxidative phosphorylation; Mfn1 and 2, mitofusin 1 and 2; Opa1, optic atrophy 1; L-Opa1, long Opa1; S-Opa1, short Opa1; Drp1, dynamin-related protein 1; POMC, proopiomelanocortin; ROS, reactive oxygen species; OCR, oxygen consumption rate; GLUT, glucose transporter; T2D, type 2 diabetes; mdivi-1, mitochondrial division inhibitor 1; ATP, adenosine triphosphate; IRS, insulin receptor substrate 1; AKT, serine/threonine-specific protein kinase*.

In particular, type 2 diabetes (T2D) is related to the reduced expression of **Mfn2**, which depresses oxidative phosphorylation (OXPHOS) and impairs mitochondrial fusion in skeletal muscle (46, 76). Also, liver and skeletal muscle deletion of Mfn2 in mice both result in fragmented mitochondrial networks and numerous metabolic abnormalities, including glucose intolerance and enhanced hepatic gluconeogenesis (47, 54). These confirm the crucial regulatory role of Mfn2 in insulin signaling and glucose homeostasis associated with obesity and T2D. Besides, it was first reported that proopiomelanocortin (POMC)–specific ablation of Mfn2 results in endoplasmic reticulum (ER) stress–induced leptin resistance and decreased energy expenditure for protection against obesity (49). Moreover, Mfn1 deficiency leads to a highly fragmented mitochondrial network and enhanced mitochondrial respiration capacity in the myocardium (53) and liver (54) in diabetes. Interestingly, the liver from Mfn1 knockout mice demonstrates a preference for using lipids as the main energy source and a more active complex I to protect against insulin resistance (54). Also during the fast-to-fed transition, mice lacking Mfn1 in POMC neurons exhibit defective mitochondrial architecture and flexibility, which results in defective insulin secretion and abnormal glucose homeostasis by pancreatic β cells (56). Mfn2, but not Mfn1, deletion in brown adipose tissue (BAT) remodels the mitochondrial dysfunction, leading to an increase in insulin sensitivity and resistance to obesity (51, 52, 77). These observations have implicated a crucial role of mitofusins in the control of mitochondrial energy metabolism and insulin resistance. However, further studies should determine the distinct regulatory mechanisms of Mfn1 and Mfn2 targeting mitochondrial metabolism in diabetes and obesity.

The expanding evidence also displays the involvement of **Opa1** in obesity and diabetes. With regard to adiposity, Opa1 has been reported to be involved in regulating lipolysis by anchoring a pool of PKA that phosphorylates perilipin and thereby triggers lipolysis *in vitro* (57) and *in vivo* (61). Further studies show the translocation of Opa1 from mitochondria to lipid droplets during human adipocyte differentiation (78). Proteases YME1L1 and Oma1 can cleave L-Opa1 into S-Opa1 (79). The deficiency of Oma1 perturbs the mitochondrial fusion–fission equilibrium, thereby reducing OXPHOS, enhancing fatty acid oxidation and decreasing energy expenditure, all of which collaboratively induce obesity in mice (79). Moreover, Opa1 deletion in pancreatic β cells impairs glucose-stimulated adenosine triphosphate (ATP) production and insulin secretion, which subsequently develops into hyperglycemia (62). Also, insulin increases Opa1 levels and promotes mitochondrial fusion in cardiomyocytes, which enhances OXPHOS, whereas Opa1 deletion suppresses the insulin-stimulated ATP synthesis (63). A recent study reported that sedentary but not active humans display an age-related decline in Opa1, which is associated with muscle loss (58). Acute muscle-specific deletion of Opa1 induces muscle inflammation, ER stress, and secretion of fibroblast growth factor 21 (FGF21) (58–60). Apart from these, a recent study addressed the physiological relevance of the concomitant impairment of mitochondrial fusion and fission machinery (80). These confirm the close relationship between Opa1 and mitochondrial insulin-stimulated energy metabolism, which provides a basis for its regulatory role in obesity and diabetes. Therefore, it is urgent to elucidate how Opa1 processing regulates mitochondrial metabolism in obesity and diabetes.

The mitochondrial fission machinery protein **Drp1** is also involved in obesity and T2D through its effect on the rate of mitochondrial oxidative metabolism. Drp1-mediated mitochondrial fission results in mitochondrial fragmentation along with decreased ATP content, which further leads to reduced insulin-mediated glucose uptake in human skeletal muscle (66) and significant loss of glucose-stimulated insulin secretion in mouse pancreatic β cells (69). Also in pancreatic β cells, Drp1 deletion largely impairs glucose-stimulated insulin secretion without affecting oxygen consumption and intracellular calcium mobilization (69). On the contrary, Drp1 regulates insulin secretion *via* glucose-fueled respiration, and pyruvate completely rescues the impaired insulin secretion of fission-deficient β cells (67). These data appear inconsistent, and the regulatory machinery of Drp1 affecting insulin secretion requires further clarification. When used to treat mice, mitochondrial division inhibitor 1 (mdivi-1), a Drp1 inhibitor, rescues tubular mitochondrial network and membrane potential, decreases oxidative stress, and increases insulin-mediated glucose uptake in diabetes-susceptible cybrid cells (68). However, the inhibition of Drp1 by mdivi-1 has no effect on reactive oxygen species (ROS) production, lipid accumulation, or the expression of adipogenic-related proteins in differentiated 3T3-L1 adipocytes (75). In contrast, overexpression of Drp1 decreases mitochondrial network formation and increases mitochondrial ROS, subsequently suppressing the insulin receptor substrate 1 (IRS1) – serine/threonine-specific protein kinase (AKT) pathway and glucose transporter (GLUT) translocation stimulated by insulin (68). Also, the depressed phosphorylation of Drp1 at S616 in skeletal muscle is negatively correlated with enhancement in fat oxidation and insulin sensitivity (66), which is further supported by the observation that Drp1 deletion induced aberrant mitochondrial fission, improving insulin signaling and systemic insulin sensitivity in skeletal muscle of obese mice (70). Moreover, Drp1 deficiency reduces fat mass, induces ER stress, and promotes energy expenditure through increasing the expression of FGF21 in the liver, which helps mice fed with a high-fat diet to be protected against obesity (72). Mice lacking Drp1 in skeletal muscle develop a lethal mitochondrial myopathy, and this muscle-specific Drp1 deletion impairs mitochondrial function, including the reduced activities of complexes I and III (71). In contrast, Drp1 overexpression in skeletal muscle displayed a drastic impairment in postnatal muscle growth, with reorganization of the mitochondrial network and reduction of mitochondrial DNA (mtDNA) quantity, without the deficiency of mitochondrial bioenergetics (64). Muscle-specific Drp1 heterozygote mice have impaired muscle endurance and running performance (65). The impaired mitochondrial remodeling induced by Drp1 in skeletal muscle is also associated with derangements in metabolism and insulin sensitivity (65, 66). Additionally, inducible deletion of Drp1 in POMC neurons (73) and the dorsal vagal complex (DVC) (74) increases ROS content, leptin sensitivity, and glucose responsiveness. Thus, Drp1-mediated mitochondrial fission may underlie the pathogenesis of insulin resistance in obesity and T2D. However, further studies are required to explore potential mechanisms for therapeutic interventions.

## Mitochondrial Fusion and Fission Shape Cancer Metabolism

Most studies of the cancer-related mitochondrial dynamics focus on their functions in apoptosis, which have been well-reviewed (1, 6). Therefore, here we focus on energy metabolism in cancer, another important function of mitochondria (Table 2). Cancer metabolism also has a history of about 100 years. In 1925, Dr. Otto Warburg first described that cancer cells tend to favor metabolism *via* aerobic glycolysis rather than the much more efficient OXPHOS pathway, which is widely known as the “Warburg effect” (93–96). Although Dr. Warburg hypothesized that dysfunctional mitochondria were the source of anaerobic glycolysis, more recent studies found that cancer cells often have intact mitochondrial metabolism (97, 98).

**Table 2:** The role of GTPases in cancer metabolism.

**GTPase**	**Approach**	**Cancer type**	**Mitochondrial metabolism**	**References**
Mfn2	Knockdown	Lung	Induce sucrose metabolism	(81)
	Knockdown	Ovarian	Reduce OCR	(82)
	Knockdown	Breast	Reduce OCR	(83)
	Knockdown	Melanoma	Reduce OCR	(84)
	Knockdown	Pancreatic	Enhance mitochondrial metabolism	(85)
	Knockout	Liver	Reduce OXPHOS; enhance lipid metabolism	(86)
Mfn1/2	Knockdown	iPSCs	Reduce ATP content	(87)
	17β-estradiol	Breast	Increase ATP content	(88)
Opa1	Overexpression	MAFs	Enhance ATP synthase oligomerization	(89)
	IF1 mutation	Cervical	Reduce OCR	(90)
Drp1	S637A mutation	Liver	Reduce OCR	(91)
	Knockdown	Brian	Reduce OCR	(92)
	Knockdown	Melanoma	Increase OCR	(42)
	Knockdown	Kidney	Reduce maximal OCR	(41)
	Knockdown	Melanoma	Maintain OCR	(84)
	mdivi-1	Melanoma	Maintain OCR	(84)
	mdivi-1	Breast	Increase OCR	(83)

*iPSCs, induced pluripotent stem cells; MAFs, mouse adult fibroblasts, IF1, inhibitory factor 1*.

Mitochondrial morphology undergoes a dynamic balance between fusion and fission in response to the altered extracellular nutrient level (5). Meanwhile, cancer metabolism is also known for its flexibility to the surrounding nutrient composition (99), and cancer cells acquire different metabolic programs corresponding to their development stages. Rapid proliferating cancer cells have a high glycolytic activity and fatty acid synthesis rate, which provide the building blocks for cell growth (100). Metastatic cancer cells have high oxidative metabolism, which maintains the energy need for mobility (101, 102). In general, oxidative metabolism is less active in the fragmented mitochondria when compared to the tubular mitochondria. Limited mitochondrial oxidation preserves glycolytic intermediates, which can be used as the building blocks for cancer cell proliferation. The highly activated glycolysis has been linked to mitochondrial fission in many types of cancer, including lung cancer (103), metastatic breast cancer (104), ovarian cancer (105), colorectal cancer (106, 107), pancreatic cancers (41), and melanoma (42). In the meantime, active oxidative metabolism has also been connected to mitochondrial fusion in some metastatic cancer models, including pancreatic cancer (108).

**Mfn1/2** shows altered expression in human tumors (109, 110). Lower Mfn2 expression is observed in breast cancer (111), lung cancer (103), urinary bladder cancers (112), hepatocellular carcinoma (113), colorectal cancer (106), and gastric cancer (114), compared to the normal tissue. In line with the lower Mfn2 expression in cancer, several studies demonstrate that Mfn2 overexpression inhibits cancer cell proliferation and colony formation and weakens the invasion and migratory ability (106, 114–116). It is worth noting that the higher expression of Mfn2 has also been reported in lung adenocarcinoma tissues as compared to adjacent normal tissues, and Mfn2 knockdown results in impaired cancer cell proliferation (81). In ovarian cancer (82), breast cancer (83), and melanoma (84), Mfn2 knockdown suppresses oxygen consumption rate (OCR). In pancreatic cancer cells (PANC-1), Mfn2 knockdown enhances mitochondria-dependent energy metabolism by promoting activity of electron transport chain complexes (85). Besides, Mfn2 knockdown induces mitochondrial biogenesis and elevates OXPHOS in Ras-transformed mice fibroblasts (117). A recent study reported that liver-specific ablation of Mfn2 in mice provoked inflammation, triglyceride accumulation, fibrosis, and liver cancer (86). As both knockdown and overexpression of Mfn2 impair cancer cell survival in certain cancer types, further studies need to further explore the different roles of Mfn2 in various tumor types.

**Opa1** and its proteolytic processing are important to maintain mitochondrial fusion. Studies have displayed a significant decrease in the expression of Opa1 along with high mitochondrial fragmentation in hepatocellular carcinoma (118). Tumor suppressor p53 is involved in Oma1-mediated L-Opa1 processing and mitochondrial fragmentation in ovarian and cervical cancer cells (119), the role of which is further supported by the close relationship between S-Opa1 and the p53 signaling pathway (120). Interestingly, in mouse adult fibroblasts (MAFs), Opa1 interacts with mitochondrial F1F0-ATP synthase, favors ATP synthase oligomerization, and finally, protects mitochondria from respiratory chain inhibition by modulating crista shape (89). Subsequent studies have discovered that the ATPase inhibitory factor 1 (IF1), a mitochondrial protein, prevents apoptotic remodeling of mitochondria by inhibiting Oma1 activation and Opa1 processing in HeLa cells, which further decreases OCR and intracellular ATP synthesis (90). Furthermore, Oma1 is dependent on the IF1 protective activity against apoptotic processing of Opa1 in HeLa cells (90). These observations display an essential role of ATP synthase in Opa1 processing to ensure normal mitochondrial bioenergetics. Since Opa1 processing complicates its function in maintenance of mitochondrial metabolism, future studies are required to focus more on the distinct regulatory machinery of Opa1 and its processing involved in mitochondrial metabolism within various cancer cells. This will provide insights into anti-tumor pharmaceutical therapy targeting Opa1-regulating mitochondrial metabolism.

**Drp1** expression and activity have been associated with mitochondria fragmentation, which facilitates glycolysis in cancer cells. High Drp1 expression has been detected in lung cancer (103), breast cancer (104), thyroid cancer (121), glioblastoma (92), and hepatocellular carcinoma (91). In 2015, two back-to-back studies demonstrated the essential role of Drp1 activation in MAPK-driven tumor growth (41, 42). Moreover, Drp1 can be activated and phosphorylated by extracellular signal-regulated kinase (ERK1/2) on S616, which is required for Ras-induced transformation in MEFs (42). This S616 phosphorylation and mitochondrial fragmentation is enhanced in human pancreatic cancer (41) and brain tumor initiating cells (BTICs) (92). Similarly, the inhibition of Erk signaling promotes mitochondrial fusion and increases mitochondrial metabolism but reduces the Drp1 activity in A375, SK-MEL-28, and BT-474 cells (42). On the contrary, the reduced expression of Drp1 exerts different effects on oxidative metabolism in different cells. For instance, Drp1 knockdown increases OCR and promotes ATP production in Ras-transformed MEFs and the SK-MEL-28 cells (42). However, Drp1 knockdown decreases the maximal OCR in the HRas-transformed HEK-TtH cells (41) and in the T387 BTICs (92), which further prevents cell growth and tumor formation. A recent study shows that in lung cancer cells, sirtuin 4 (SIRT4, an nicotinamide-adenine dinucleotide+ (NAD+)-dependent protein deacetylase) inhibits Drp1 phosphorylation at the S616 site and weakens Drp1 recruitment to the mitochondrial membrane *via* an interaction with Fis1 (122). Besides, Drp1 can also be phosphorylated on S637, which subsequently represses its activity and mitochondrial translocation. Particularly, S637-mediated mitochondrial elongation under energy stress is essential for hepatocellular carcinoma cell survival both *in vitro* and *in vivo* (91). Interestingly, lung cancer cell lines have higher S616 and lower S637 phosphorylation, when compared to human bronchial epithelial cell (hBEC) (103).

**mdivi-1** is widely used as a Drp1 inhibitor (30, 123). In 2008, Dr. Nunnari's group first utilized mdivi-1 to study mitochondrial dynamics (45), and most of the studies have followed this application of mdivi-1 to examine the metabolic role of Drp1. mdivi-1 treatments result in both increased and decreased OCR, depending on different cell types. In most conditions, mdivi-1 treatment induces mitochondrial fusion and increases mitochondrial activity, which further enhances OCR (124). In MDA-MB-231 cells and H1299 cells, mdivi-1 treatment significantly depresses OCR, without changing the cellular ATP level (125). Interestingly, a study shows that mdivi-1 represses OCR to a similar degree in both wild-type and Drp1 knockout MEF cells (126). This Drp1-independent role of mdivi-1 is further supported by the finding that mdivi-1 works as mitochondrial complex I inhibitor, to induce ROS and repress OCR (126). In the highly oxidative estrogen receptor–positive breast cancer cells, mdivi-1 reduces cellular mitochondrial bioenergetics and increases glycolysis without any influence on mitochondrial morphology (127). Overall, mdivi-1 has potential therapeutic avenues in suppressing tumor progression. Further studies are required to explore its effects on the oxidative metabolic alterations in cancer.

## Summary

**Figure 1:**
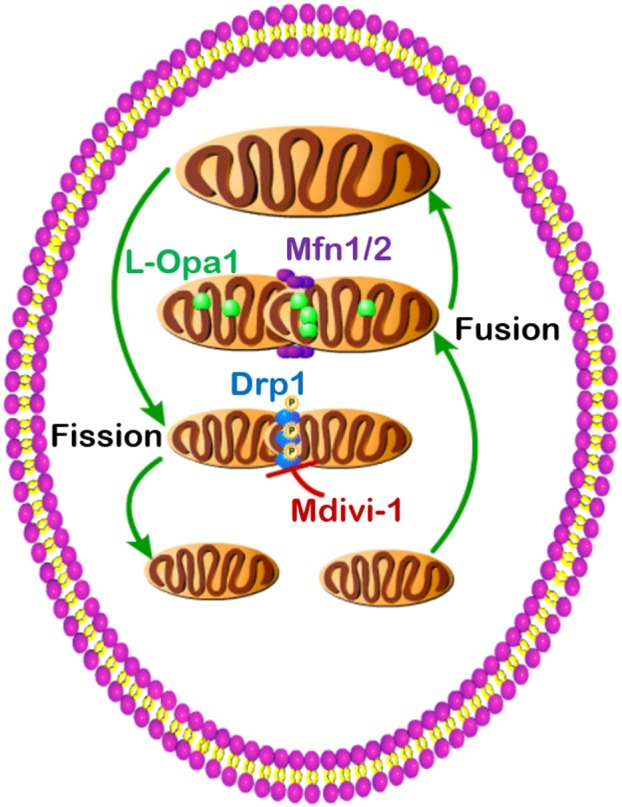
Dysregulated mitochondrial dynamics and metabolism in obesity, diabetes, and cancer.

Dysregulated metabolism is a common feature of the metabolic diseases, including obesity, diabetes, and cancer, which can be regulated by mitochondrial dynamics (Figure 1). Obese and diabetic patients have pathology in multiple tissues, such as fat, muscle, liver, kidney, and pancreas. Cancer patients also have tumors from different origins, and all cancer types undergo different stages. Moreover, the specific mitochondrial dynamics under these pathology conditions are in response to different metabolic states, and thus, it is important to discuss the mitochondrial dynamics and metabolism in a context-dependent manner. For example, mild nutrient deprivation induces mitochondrial fusion and increases energy-producing efficiency to compensate the energy demand for growth, while severe nutrient deprivation drives mitochondrial fission and promotes cell apoptosis to preserve energy for neighbor cells. As demonstrated here, the GTPases (Mfn1, Mfn2, Opa1, and Drp1) have strong regulatory effects in balancing mitochondrial fusion and fission, and these GTPases control the aforementioned metabolic diseases predominantly through orchestrating oxidative metabolism. It is essential to explore how the GTPases orchestrate metabolic alterations under certain nutrient environments. Since mitochondrial morphology undergoes a dynamic process balanced by the above GTPases, knockdown and knockout of these result in abnormal chronical inhibition and can potentially cause indirect metabolic effects. Therefore, it is important to develop new chemical inhibitors to acutely block the function of these GTPases. Oxygen consumption is only a small fraction in metabolism, and more direct metabolomics and metabolic flux analyses will provide a clearer picture of the mitochondrial dynamics and metabolism in obesity, diabetes, and cancer.

## Author Contributions

LJ designed the review. WD made the figure and tables. WD and LJ wrote the review.

### Conflict of Interest Statement

The authors declare that the research was conducted in the absence of any commercial or financial relationships that could be construed as a potential conflict of interest.
